# Modelling Optimal Control of In-Host HIV Dynamics Using Different Control Strategies

**DOI:** 10.1155/2018/9385080

**Published:** 2018-06-04

**Authors:** Purity Ngina, Rachel Waema Mbogo, Livingstone S. Luboobi

**Affiliations:** Strathmore Institute of Mathematical Sciences, Strathmore University, P.O. Box 59857-00200, Nairobi, Kenya

## Abstract

HIV is one of the major causes of deaths, especially in Sub-Saharan Africa. In this paper, an in vivo deterministic model of differential equations is presented and analyzed for HIV dynamics. Optimal control theory is applied to investigate the key roles played by the various HIV treatment strategies. In particular, we establish the optimal strategies for controlling the infection using three treatment regimes as the system control variables. We have applied Pontryagin's Maximum Principle in characterizing the optimality control, which then has been solved numerically by applying the Runge-Kutta forth-order scheme. The numerical results indicate that an optimal controlled treatment strategy would ensure significant reduction in viral load and also in HIV transmission. It is also evident from the results that protease inhibitor plays a key role in virus suppression; this is not to underscore the benefits accrued when all the three drug regimes are used in combination.

## 1. Introduction

There is an ever-changing need for new and useful treatment regimes that will provide assistance and relief in all aspects of the human condition. Subsequently, many researchers have embarked on the journey of analyzing the dynamics of various diseases affecting mankind with the aim of improving control and effect and finally eradicating the diseases from the population. Modelling and numerical simulations of the infectious diseases have been used as tools to optimize disease control. This is due to the fact that medical community has insufficient animal models for testing efficacy of drug regimes used in controlling infections. Human immunodeficiency virus (HIV) is one of the major problems that researchers have been working on for over three decades. According to the Joint United Nations Programme on HIV and AIDS (UNAIDS), there were 36.7 million people living with HIV/AIDS in 2016, 1.6 million of which live in Kenya [1]. Nonetheless, many treatment regimes for HIV have been approved by the US Food and Drug Administration. Highly Active Antiretroviral Therapy (HAART) is the latest combination in use for HIV treatment in most countries. HAART has been proven to be highly effective in viral suppression, prolongs life of the infected person, and also reduces the rate of HIV transmission. However, even over three decades since the first HIV cases were reported, the virus had no cure and hence various control methods for HIV/AIDS have been recommended. These controls range from preventive measures to treatment regimes. Preventive measures aim at reducing the number of new HIV infections, while treatment regimes target the already infected persons to increase their life expectancy and reduce the rate of HIV transmission. Various treatment strategies are still the subject of many ongoing clinical trials that are investigating their benefits versus risks aimed at determining the most optimal treatment for HIV. Unfortunately, various host-pathogen interaction mechanisms during HIV infection and progression to AIDS are still unknown. Consequently, many questions like which is the best combination, when is the best time to start treatment, and how the treatment should be administered are yet to be answered fully.

Mathematical modelling is one of the many important tools used in understanding the dynamics of disease transmission. It is also used in developing guidelines important in disease control. In HIV, mathematical models have provided a framework for understanding the viral dynamics and have been used in the optimal allocation of the various interventions against the HIV virions [2–4]. A fundamental goal of developing and applying the aforementioned mathematical models of HIV is to influence treatment decisions and construct better treatment protocols for infected patients. Most of the modern mathematical models that have been developed apply the optimal control theory. Optimal control theory is a branch of mathematics developed to find optimal ways of controlling a dynamical system [5]. It has been applied by mathematicians to assist in the analysis of how to control the spread of infectious diseases. The results are used in making key decisions that involve complex biological mechanism. In particular, it is used to determine the best dosage for various available vaccines or treatment in use for controlling infection. For instance, Gaff and Schaefer [6] applied optimal theory in evaluating mitigation strategy that would be highly effective in minimizing the number of people who get infected by an infection. The study applied both vaccinations and treatment as control variables for their various model. The results indicated that as much as treatment is paramount in controlling any infection, the most optimal method would be the combination of the two interventions. Furthermore, Bakare et al. [7] applied optimal control in an SIR model. The study illustrated the use of optimal control theory in establishing the optimal educational campaign and treatment strategies that would minimize the population of the infected persons as well as cutting the cost of controlling the various diseases. The results indicated that, for controlling infection, it is important to target the uninfected populations and apply measures that will prevent them from getting the infection.

In the literature, optimal control theory has been applied in the analysis of in-host HIV dynamics as well as in population-based HIV models. For instance, Yusuf and Benyah [14] applied optimal theory on HIV population model. The study aimed at determining the best method of controlling the spread of HIV/AIDS within a specified time frame. The study considered three control variables, that is, safe sex, education, and ARTs. The numerical results of the objective function for the model indicated that safe sex practice and early initiation of ARTs are the most optimal ways of mitigating the spread of HIV/AIDS. The study established that if the aforementioned strategies are well implemented, this would lead to an HIV-free nation in 10 years. In addition, for in-host model, optimal control theory has been applied in the search for optimal therapies for HIV infection.

Drugs such as fusion inhibitors (FIs), reverse transcriptase inhibitors (RTIs), and protease inhibitors (PIs) have been developed and applied in the various optimal control problems. Srivastava et al. [15] analyzed an initial infection model with reverse transcriptase inhibitors (RTIs). The study argued that, through the use of RTIs, an infected cell reverts back to susceptibility. However, this is unlikely since once a CD4^+^ T-cell is infected, it cannot recover. The only possible way is for it to remain latently infected but fail to produce infectious virus, since RTIs inhibit the reverse transcription process. Hattaf and Yousfi [16] analyzed two optimal treatments of HIV infection model. The study aimed at measuring the efficiency of RTIs and PIs. This was done by maximizing objective function aimed at increasing the number of the uninfected cells, decreasing the viral load, and minimizing the treatment cost. The results indicated that use of therapy is important in HIV control. It is also important to note that the study included two types of viruses, that is, the infectious virus and the noninfectious virus. Noninfectious virus is due to the use of PIs as a treatment regime.

Karrakchou et al. [17] applied optimal control theory on HIV. Like Hattaf and Yousfi [16], the study applied the two control strategies, that is, RTIs and the PIs. However, the study failed to put into account both the latently infected cells and the noninfectious virus that results due to the use of RTIs and PIs, respectively. Failure to include such important variables in the model underscores the adequacy of the model in representing the actual HIV in-host mechanism. In addition, Arruda et al. [12] applied optimal control theory in HIV immunology. The study used two control variables in fighting HIV with the inclusion of the CD8^+^ T-cells. However, the study has some shortcomings; for instance, the study suggested that activated CD8^+^ T-cells kill the HIV virions and also the infected cells. This is not the scenario, since the activated CD8^+^ T-cells are only able to kill infected CD4^+^ T-cells which in turn reduce the population of the HIV virions. Unfortunately, even with the aforementioned work done on HIV, the implementation of some of the recommendations has been proven to be inefficient and in most cases not economically viable, especially to the developing countries.

As per the literature cited, it is clear that as much as ARTs have been used for viral suppression, the optimal treatment schedule necessary to maintain low viral load is always an approximation. Until the time when HIV cure is found, physicians will try as much as possible to apply the control strategy that will inhibit viral progression while simultaneously holding the side effects of treatment to a minimum. Most of the treatment regimes have many side effects that must be maintained at a low level. For example, long-term use of protease inhibitors is associated with insulin intolerance, cholesterol elevation, and the redistribution of body fat. Therefore, there is a need to establish the optimal treatment strategy, that is, the one which both maximizes the patient's uninfected CD4^+^ T-cells and minimizes the harmful side effects due to the drugs.

This study has addressed some of the shortcomings noted from the in-host HIV dynamics models by applying three control variables representing the three drug regimes on the market, that is, the fusion inhibitor, reverse transcriptase inhibitors, and the protease inhibitors, in the in vivo HIV model. In addition, the study has incorporated the CD8^+^ T-cells in the model. For the analysis, the study will apply optimal control theory together with Pontryagin's Maximum Principle in solving the objective function with the aim of establishing the optimal treatment strategy.

## 2. Model Formulation

### 2.1. Model Description

In order for us to carry out optimal control processes, it is paramount to formulate a model that describes the basic interaction between the HIV virions and the body immune system. We develop a mathematical model for HIV in-host infection with three combinations of drugs. We define seven variables for the model as follows: susceptible CD4^+^ T-cells (*T*), latently infected CD4^+^ T-cells (*I*_*l*_), infected CD4^+^ T-cells (*I*), HIV infectious virions (*V*), noninfectious HIV virions (*V*_*n*_), CD8^+^ T-cells (*Z*), and the activated CD8^+^ T-cells (*Z*_*a*_).

The parameters for the model are as follows. The susceptible CD4^+^ T-cells are produced from the thymus at a constant rate *λ*_*T*_, die at a constant per capita rate *μ*_*T*_, and become infected by the HIV virions at the rate *χTV*. However, due to the use of fusion inhibitor (*u*_1_) which prevents the entry of the HIV virions into the CD4^+^ T-cells, a fraction *u*_1_*χVT* reverts back to susceptible class. In addition, when the infected CD4^+^ T-cells are exposed to the HIV virions in presence of reverse transcriptase inhibitor (*u*_2_), the HIV virions RNA may not be reverse-transcribed. This results in a proportion *u*_2_*χVT* of the infected cells becoming latently infected. The infected cells are killed by the CD8^+^ T-cells at the rate *α* and they die naturally at the rate *μ*_*I*_, whereas latently infected cells die at the rate *μ*_*I*_*l*__. This study assumes that the latently infected cells will die naturally and have no possibility of producing infectious virions nor becoming activated to become infectious. However, if the protease inhibitor (*u*_3_) is used as a treatment strategy, it inhibits the production of protease enzyme, which is necessary for production of mature HIV virions. This therefore means that we have two kinds of HIV virions produced from infected CD4^+^ T-cells, that is, the infectious HIV virions and the immature noninfectious virions. The infectious HIV virions are produced at the rate (1 − *u*_3_)*ϵ*_*V*_ and die at the rate *μ*_*V*_, while the noninfectious HIV virions are produced at the rate *u*_3_*ϵ*_*V*_ and die at the rate *μ*_*V*_*n*__. Furthermore, the CD8^+^ T-cells are produced naturally from the thymus at the rate *λ*_*Z*_, they die naturally at the rate *μ*_*Z*_, and they can also be activated to kill the infected cells at the rate *β*. The activated CD8^+^ T-cells die naturally at the rate *μ*_*Z*_*a*__. It is very important to point out that the CD8^+^ T-cells are activated to kill the infected CD4^+^ T-cells and not the virus as suggested by Arruda et al. [12].

The summary for the model description is given as follows. The variables, parameters, and the control variables for the in-host model are described in Tables 1, 2, and 3, respectively.

**Figure 1:**
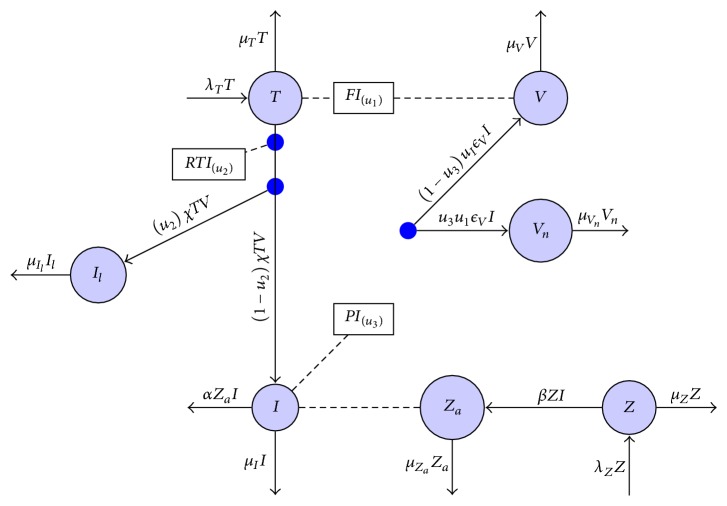
A compartmental representation of the in vivo HIV dynamics with therapy.

From Figure 1 and the description above, we derive the following system of ordinary differential equations to describe the in vivo dynamics of HIV:

(1) dTdt=λT−μTT−1−u1tχTV,dIdt=1−u2tχTV−μII−αIZa,dIldt=u2tχTV−μIlIl,dVdt=1−u3tϵVμII−μVV,dVndt=u3tϵVμII−μVnVn,dZdt=λZ−μZZ−βZI,dZadt=βZI−μZaZa.

## 3. Optimization Process

Control efforts are carried out to limit the spread of the disease and, in some cases, to prevent the emergence of drug resistance. Optimal control theory is a method that has been widely used to solve for an extremum value of an objective functional involving dynamic variables. In this section, we consider optimal control methods to derive optimal drug treatments as functions of time. The control variables as used in (1) are described as follows. The control *u*_1_ represents the effect of fusion inhibitors, which are the drugs that protect the uninfected CD4^+^ T-cells by preventing the entry of the virus into the CD4^+^ T-cells membrane. The control variable *u*_2_ simulates the effect of reverse transcriptase inhibitors. These drugs hinder the reverse transcription process. The third control variable *u*_3_ simulates the effect of protease inhibitors, which prevent the already infected cells from producing mature infectious virions. The aforementioned controls represent effective chemotherapy dosage bounded between 0 and 1. The situation *u*_1_(*t*) = *u*_2_(*t*) = *u*_3_(*t*) = 1 represents total efficacy of the fusion inhibitors, reverse transcriptase inhibitors, and protease inhibitors, respectively, and *u*_1_(*t*) = *u*_2_(*t*) = *u*_3_(*t*) = 0 represents no treatment. It is worth noting that the aforementioned control variables are bounded Lebesgue-integrable functions. The study aims at maximizing the levels of the healthy CD4^+^ T-cells, as well as the levels of the CD8^+^ T-cells (*Z*), while minimizing the viral load (*V*) and at the same time keeping cost and side effects of treatment at a minimum. With the above description, the following objective function (2) needs to be maximized:

(2) Ju1t,u2t,u3t=12∫0Tfw1Tt+w2Zt−w3Vt−A1u12−A2u22−A3u32dt

subject to the ordinary differential equations given in model (1).

*T*(*t*), *Z*(*t*), and *V*(*t*) are the solutions of the ODEs (1). The quantities *w*_1_ and *w*_2_ represent the cost associated with maximizing the number of CD4^+^ T-cells and the CD8^+^ T-cells, respectively, while *w*_3_ represents the cost associated with minimizing the viral load. In addition, *A*_1_, *A*_2_, and *A*_3_ are nonnegative constants representing the relative weights attached to the current cost of each treatment regime and *T*_*f*_ is a fixed terminal time of the treatment program subject to the ordinary differential equations described in model (1). This study assumes that the cost of controls is of quadratic form. Furthermore, it is also based on the fact that there is no linear relationship between the effect of treatment on CD4^+^ T-cells and CD8^+^ T-cells and the HIV virions. Consequently, *u*_1_, *u*_2_, and *u*_3_ are Lebesgue-integrable; that is, they are piecewise continuous and integrable. The fundamental aim of this therapeutic strategy is to maximize the objective functional defined in (2) by increasing the number of the uninfected CD4^+^ T-cells and the CD8^+^ T-cells, decreasing the viral load (*V*), and minimizing the harmful side effects and cost of treatment over the given time interval [0, *T*_*f*_]. Therefore, we aim at determining the optimal controls *u*_1_^*∗*^, *u*_2_^*∗*^, and *u*_3_^*∗*^ such that

(3) Ju1∗t,u2∗t,u3∗t=max⁡Ju1t,u2t,u3t:u1,u2,u3∈U,

where *U* is a set of all measurable controls defined by

(4) U=u=u1,u2,u3:ui measurable,  0≤uit≤1,  t∈0,Tf.

In the next section, we show the existence of an optimal control for system (1) and later derive the optimality system. This study will employ Pontryagin's Maximum Principle.

## 4. Characterization of the Optimal Control

The necessary conditions that an optimal control must satisfy come from Pontryagin's Maximum Principle [5].

**Theorem 1 .**

Suppose that the objective function

(5) Ju1t,u2t,u3t=12∫0Tfw1Tt+w2Zt−w3Vt−A1u12−A2u22−A3u32dt

is maximized subject to the controls and state variables given in model (1) with

(6) T0=T0,I0=I0,Il0=Il0,V0=V0,Vn0=Vn0,Z0=Z0,Za0=Za0.

Then there exist optimal controls (*u*_1_^*∗*^, *u*_2_^*∗*^, *u*_3_^*∗*^ ∈ *U*) such that

(7) Ju1∗t,u2∗t,u3∗t=max⁡Ju1t,u2t,u3t:u1,u2,u3∈U.

**Proof**

The existence of the solution can be shown using the results obtained in Fleming and Rishel [18], since

(1) the class of all initial conditions with controls *u*_1_, *u*_2_, and *u*_3_ in the control set *U* are nonnegative values and are nonempty, where *u*_*i*_, *i* = 1,2, 3, is a Lebesgue-integrable function on [0, *T*_*f*_],
(2) the right-hand side of system (1) is bounded by a linear function of the state and control variables,
by definition, each right-hand side of system (1) is continuous and can be written as a linear function of *U* with coefficients depending on time and state. Furthermore, all the state and control variables *T*, *I*, *I*_*l*_, *V*, *V*_*n*_, *Z*, *Z*_*a*_, *u*_1_, *u*_2_, and *u*_3_ are bounded on [0, *T*_*f*_],
(3) by definition, the control set *U* is convex and closed.A set *K* ∈ *ℝ*^⋉^ is said to be a convex set if and only if
  (8) $$\begin{array}{c} \lambda x+\left(\;1-\lambda \;\right)y\in K \end{array}$$
  for all *x*, *y* ∈ *K* and all *λ* ∈ [0,1],
this condition is satisfied by the control set *U*,
(4) the integrand which is (1/2)(*A*_1_*u*_1_^2^ + *A*_2_*u*_2_^2^ + *A*_3_*u*_3_^2^) of the objective functional is concave on *U*,
(5) there exist constants *b*_1_ > 0,  *b*_2_ > 0, and *β* > 1 such that the integrand of the objective function *J*(*U*, *t*) is bounded by *L*(*t*, *T*, *V*, *V*_*n*_, *I*, *I*_*l*_, *Z*, *Z*_*a*_, *u*_1_, *u*_2_, *u*_3_) ≤ *b*_2_ − *b*_1_(|*u*_1_|^2^ + |*u*_2_|^2^ + |*u*_3_|^2^)^*β*/2^,
this implies that
  (9) w1Tt+w2Zt−w3Vt−A1u12−A2u22−A3u32≤b2−b1u12+u22+u32,
  where *b*_1_ depends on the upper bound on *T*, *Z*, *V* while *b*_1_ > 0 since *A*_1_, *A*_2_, *A*_3_ > 0 according to the definition.

Since all the above conditions are satisfied, we conclude that there exist optimal controls *u*_1_^*∗*^, *u*_2_^*∗*^, and *u*_3_^*∗*^.

## 5. Necessary Conditions of the Control

We now proceed by applying Pontryagin's Maximum Principle [5]. We begin by defining Lagrangian (Hamiltonian augmented):

(10) LT,I,Il,V,Vn,Z,Za,λ1,λ2,λ3,λ4,λ5,λ6,λ7,u1,u2,u3=w1T+w2Z−w3V−A1u12−A2u22−A3u32+λ1λT−μTT−1−u1tχTV+λ21−u2tχTV−μII−αIZa+λ3u2t·χTV−μIlIl+λ41−u3tϵVμII−μVV+λ5u3tϵVμII−μVnVn+λ6λZ−μZZ−βZI+λ7βZI−μZaZa+w11u1+w121−u1+w21u2+w221−u2+w31u3+w321−u3,

where *w*_*ij*_(*t*) ≤ 0 are the penalty multipliers that ensure the boundedness of the control variables *u*_1_(*t*), *u*_2_(*t*), and *u*_3_(*t*) and satisfy the following conditions:

(11) w11u1=w121−u1=0at  u1∗w21u2=w221−u2=0at  u2∗w31u3=w321−u3=0at  u3∗,

where *u*_1_^*∗*^, *u*_2_^*∗*^, and *u*_3_^*∗*^ represent the optimal controls.

Therefore, Pontryagin's Maximum Principle gives the existence of adjoint variables that are obtained by differentiating the Lagrangian given by (10) with respect to the state variables *T*, *V*, *I*, *I*_*l*_, *Z*, and *Z*_*a*_.

The adjoint variables are given by

(12) λ˙1=−∂L∂T=−w1+λ1μT+1−u1χV−λ2χV1−u2−λ3u2χV,λ˙2=−∂L∂I=λ2μI+αZa−λ4εVμI1−u3−λ5u3ϵVμI+λ6βZ−λ7βZ,λ˙3=−∂L∂Il=λ3μIl,λ˙4=−∂L∂V=w3+λ1χT1−u1−λ2χT1−u2−λ3χTu2+λ4μV,λ˙5=−∂L∂Vn=λ5μVn,λ˙6=−∂L∂Z=−w2+λ6μZ+βI−λ7βI,λ˙7=−∂L∂Za=λ2αI+λ7μZa,

where

(13) $$\begin{array}{c} \lambda_{i}\left(\;T_{f}\;\right)=0,\;i=1,\ldots ,7, \end{array}$$

are the transversality conditions.

By maximization of the Lagrangian with respect to the control variables *u*_1_, *u*_2_, *u*_3_ at the optimal controls (*u*_1_^*∗*^, *u*_2_^*∗*^, and  *u*_3_^*∗*^), we have

(14) ∂L∂u1=0,∂L∂u2=0,∂L∂u3=0.

Therefore, differentiating the Lagrangian *L* given in (10) with respect to *u*_1_ on the set *U* : *t*∣0 ≤ *u*_1_(*t*) ≤ 1, we get the following optimality equation:

(15) $$\begin{array}{c} \frac{\partial L}{\partial u_{1}}=-2A_{1}u_{I}+\chi TV\lambda_{1}+w_{11}-w_{12}=0. \end{array}$$

Let *u*_1_ = *u*_1_^*∗*^ in (15). Then, solving (15), we obtain the optimal control *u*_1_^*∗*^ as

(16) $$\begin{array}{c} u_{1}^{*}=\frac{\chi TV\lambda_{1}+w_{11}-w_{12}}{2A_{1}}. \end{array}$$

To determine an explicit expression for an optimal control *u*_1_^*∗*^ without *w*_11_ and *w*_12_, we consider the following three cases:

(1) On the set (*t*∣0 < *u*_1_^*∗*^ < 1), suppose we set *w*_11_ = *w*_12_ = 0 in (16). Then the optimal *u*_1_^*∗*^ control is given by
  (17) $$\begin{array}{c} u_{1}^{*}=\frac{\chi TV\lambda_{1}}{2A_{1}}. \end{array}$$
(2) Similarly, on the set (*t*∣*u*_1_^*∗*^ = 1), we have *w*_11_ = 0 and *w*_12_ ≥ 0; then from (16), we have
  (18) $$\begin{array}{c} u_{1}^{*}=1=\frac{\chi TV\lambda_{1}-w_{12}}{2A_{1}}. \end{array}$$
  Equation (18) can be reduced to
  (19) $$\begin{array}{c} \frac{\chi TV\lambda_{1}}{2A_{1}}\geq 1=u_{1}^{*}. \end{array}$$
  Therefore, for this set, we have
  (20) $$\begin{array}{c} u_{1}^{*}=\min\left(\;1,\frac{\chi TV\lambda_{1}}{2A_{1}}\;\right). \end{array}$$
(3) Finally, on the set (*t*∣*u*_1_^*∗*^ = 0), we have *w*_12_ = 0 and *w*_11_ ≥ 0; then from (16), we have
  (21) $$\begin{array}{c} u_{1}^{*}=0=\frac{\chi TV\lambda_{1}+w_{11}}{2A_{1}}, \end{array}$$
  which implies that
  (22) $$\begin{array}{c} \frac{\chi TV\lambda_{1}}{2A_{1}}\leq 0. \end{array}$$

Consequently, combining all the three cases given by (17), (20), and (22), we obtain the optimal control, *u*_1_^*∗*^, as follows:

(23) $$\begin{array}{c} u_{1}^{*}\left(\;t\;\right)=\left\{\begin{array}{cc} \frac{\chi TV\lambda_{1}}{2A_{1}} & if\;\;0<\frac{\chi TV\lambda_{1}}{2A_{1}}<1\\ 0 & if\;\;\frac{\chi TV\lambda_{1}}{2A_{1}}\leq 0\\ 1 & if\;\;\frac{\chi TV\lambda_{1}}{2A_{1}}\geq 1. \end{array}\right. \end{array}$$

This implies that the control *u*_1_^*∗*^(*t*) is formulated as follows:

(24) $$\begin{array}{c} u_{1}^{*}=\max\left(\;0,\min\left(\;1,\frac{\chi TV\lambda_{1}}{2A_{1}}\;\right)\;\right). \end{array}$$

We use the same argument to obtain an explicit expression for an optimal control *u*_2_^*∗*^ without *w*_21_ and *w*_22_. We differentiate the Lagrangian *L* given in (10) with respect to *u*_2_ on the set *U* : *t*∣0 ≤ *u*_2_(*t*) ≤ 1. We therefore obtain the optimality equation as

(25) ∂L∂u2=−2A2u2+χTVλ3−λ2+w21−w22=0at  u2=u2∗.

Therefore, solving (25), we obtain the optimal control *u*_2_^*∗*^ as follows:

(26) $$\begin{array}{c} u_{2}^{*}=\frac{\chi TV\left(\lambda_{3}-\lambda_{2}\right)+w_{21}-w_{22}}{2A_{2}}. \end{array}$$

According to the conditions given by (11), we derive the following distinct three cases:

(1) On the set (*t*∣0 < *u*_2_^*∗*^ < 1), we have *w*_21_ = *w*_22_ = 0 in (26). Then the optimal *u*_2_^*∗*^ control is given by
  (27) $$\begin{array}{c} u_{2}^{*}=\frac{\chi TV\left(\lambda_{3}-\lambda_{2}\right)}{2A_{2}}. \end{array}$$
(2) On the set (*t*∣*u*_2_^*∗*^ = 1), we have *w*_21_ = 0 and *w*_22_ ≥ 0; then from (26), we have
  (28) $$\begin{array}{c} u_{2}^{*}=1=\frac{\chi TV\left(\lambda_{3}-\lambda_{2}\right)+w_{22}}{2A_{2}}. \end{array}$$
  Rearranging (28) we have
  (29) $$\begin{array}{c} \frac{\chi TV\left(\lambda_{3}-\lambda_{2}\right)}{2A_{2}}\geq 1=u_{2}^{*}. \end{array}$$
  Thus, for the this set, we have
  (30) $$\begin{array}{c} u_{2}^{*}=\min\left(\;1,\frac{\chi TV\left(\lambda_{3}-\lambda_{2}\right)}{2A_{2}}\;\right). \end{array}$$
(3) Finally, on the set (*t*∣*u*_2_^*∗*^ = 0), we have *w*_22_ = 0 and *w*_21_ ≥ 0; then from (26), we have
  (31) $$\begin{array}{c} u_{2}^{*}=0=\frac{\chi TV\left(\lambda_{3}-\lambda_{2}\right)}{2A_{2}}, \end{array}$$
  which implies that
  (32) $$\begin{array}{c} \frac{\chi TV\left(\lambda_{3}-\lambda_{2}\right)}{2A_{2}}\leq 0. \end{array}$$

Consequently, combining all the three cases given by (27), (30), and (32), we obtain the optimal control *u*_2_^*∗*^ as follows:

(33) u2∗t=χTVλ3−λ22A2if  0<χTVλ3−λ22A2<10if  χTVλ3−λ22A2≤01if  χTVλ3−λ22A2≥1.

Hence, the optimal control *u*_2_^*∗*^(*t*) is formulated as follows:

(34) $$\begin{array}{c} u_{2}^{*}=\max\left(\;0,\min\left(\;1,\frac{\chi TV\left(\lambda_{3}-\lambda_{2}\right)}{2A_{2}}\;\right)\;\right). \end{array}$$

To obtain the expression for optimal control *u*_3_^*∗*^, we differentiate (10) with respect to *u*_3_ on the set *U* : *t*∣0 ≤ *u*_3_(*t*) ≤ 1 to get the following optimality equation:

(35) $$\begin{array}{c} \frac{\partial L}{\partial u_{3}}=-2A_{3}u_{3}-\varepsilon_{V}\mu_{I}I\lambda_{4}+w_{31}-w_{32}=0. \end{array}$$

Let *u*_3_ = *u*_3_^*∗*^ in (35); then we obtain the optimal control *u*_3_^*∗*^:

(36) $$\begin{array}{c} u_{3}^{*}=\frac{-\varepsilon_{V}\mu_{I}I\lambda_{4}+w_{31}-w_{32}}{2A_{3}}. \end{array}$$

(1) On the set (*t*∣0 < *u*_3_^*∗*^ < 1), we have *w*_31_ = *w*_32_ = 0 in (36). Then the optimal control *u*_3_^*∗*^ is given by
  (37) $$\begin{array}{c} u_{3}^{*}=\frac{-\varepsilon_{V}\mu_{I}I\lambda_{4}}{2A_{3}}. \end{array}$$
(2) On the set (*t*∣*u*_3_^*∗*^ = 1), we have *w*_31_ = 0 and *w*_32_ ≥ 0; then from (36), we have
  (38) $$\begin{array}{c} u_{3}^{*}=1=\frac{-\varepsilon_{V}\mu_{I}I\lambda_{4}+w_{32}}{2A_{3}}. \end{array}$$
  Equation (38) can be reduced to
  (39) $$\begin{array}{c} \frac{-\varepsilon_{V}\mu_{I}I\lambda_{4}}{2A_{3}}\geq 1=u_{3}^{*}. \end{array}$$
  Hence, for this set, we have
  (40) $$\begin{array}{c} u_{3}^{*}=\min\left(\;1,\frac{-\varepsilon_{V}\mu_{I}I\lambda_{4}}{2A_{3}}\;\right). \end{array}$$
(3) Finally, on the set (*t*∣*u*_3_^*∗*^ = 0), we have *w*_32_ = 0 and *w*_31_ ≥ 0; then from (36), we have
  (41) $$\begin{array}{c} u_{3}^{*}=0=\frac{-\varepsilon_{V}\mu_{I}I\lambda_{4}+w_{31}}{2A_{3}}, \end{array}$$
  which implies that
  (42) $$\begin{array}{c} \frac{-\varepsilon_{V}\mu_{I}I\lambda_{4}}{2A_{3}}\leq 0. \end{array}$$

Consequently, combining all the three cases given by (37), (40), and (42), the optimal control, *u*_3_^*∗*^, is characterized as

(43) $$\begin{array}{c} u_{3}^{*}\left(\;t\;\right)=\left\{\begin{array}{cc} \frac{-\varepsilon_{V}\mu_{I}I\lambda_{4}}{2A_{3}} & if\;\;0<\frac{-\varepsilon_{V}\mu_{I}I\lambda_{4}}{2A_{3}}<1\\ 0 & if\;\;\frac{-\varepsilon_{V}\mu_{I}I\lambda_{4}}{2A_{3}}\leq 0\\ 1 & if\;\;\frac{-\varepsilon_{V}\mu_{I}I\lambda_{4}}{2A_{3}}\geq 1. \end{array}\right. \end{array}$$

Therefore, the optimal control, *u*_3_^*∗*^(*t*), is formulated as

(44) $$\begin{array}{c} u_{3}^{*}=\max\left(\;0,\min\left(\;1,\frac{-\varepsilon_{V}\mu_{I}I\lambda_{4}}{2A_{3}}\;\right)\;\right). \end{array}$$

It is worth noting that the optimal controls depend on the adjoint variables *λ*_1_, *λ*_2_, *λ*_3_, and *λ*_4_, since the adjoint variables correspond to the state variables, *T*, *I*, *I*_*l*_, *V*, and the first four equations in (1) contain the control terms.

## 6. Numerical Simulation

In this section, we investigate the effect of optimal strategy on HIV by applying Runge-Kutta forth-order scheme on the optimality system. The optimality system is obtained by taking the state system together with the adjoint system, the optimal control, and the transversality conditions. The dynamical behaviour of the models in relation to various control is also studied. The optimal strategy is achieved by obtaining a solution for the state system (1) and costate system (12). An iterative scheme is explored and used to determine the solution for the optimality system. The numerical method utilized is the forward-backward sweep method that incorporates iterative Runge-Kutta fourth-order progressive-regressive schemes. The progressive scheme is used in obtaining the solutions of the state ODEs given in (1) with the initial conditions, while the regressive scheme is applied in obtaining the solutions of the adjoint system given by (12) with transversality conditions given in (13). The controls are updated at the end of each iteration using the formula for optimal controls. We continue with the iterations until convergence is achieved. This is a two-point boundary-value problem, with separated boundary conditions at times *t*_0_ = 0 and *t* = *T*_*f*_. This explains our choice in using the fourth-order Runge-Kutta scheme. For the numerical simulation, we take *T* = 310 days or 10 months. This value represents the time in which treatment is stopped. Furthermore, the values of the weight function are taken as *A*_1_ = *A*_2_ = *A*_3_ = 0.01.  Table 4 consists of the parameter values that are used in the numerical simulations of the in vivo model, while Table 5 consists of the proposed initial values of the state variables.

The initial values given in Table 5 are chosen in such a way that they reflect a patient during acute infection. This is in line with the WHO recommendations that stipulate that all people living with the HIV be put on ARTs irrespective of their CD4^+^ counts unlike in the past where the CD4^+^ count had to be less than 500 cells/mm^3^ [19].

### 6.1. Results and Discussion

**Figure 2:**
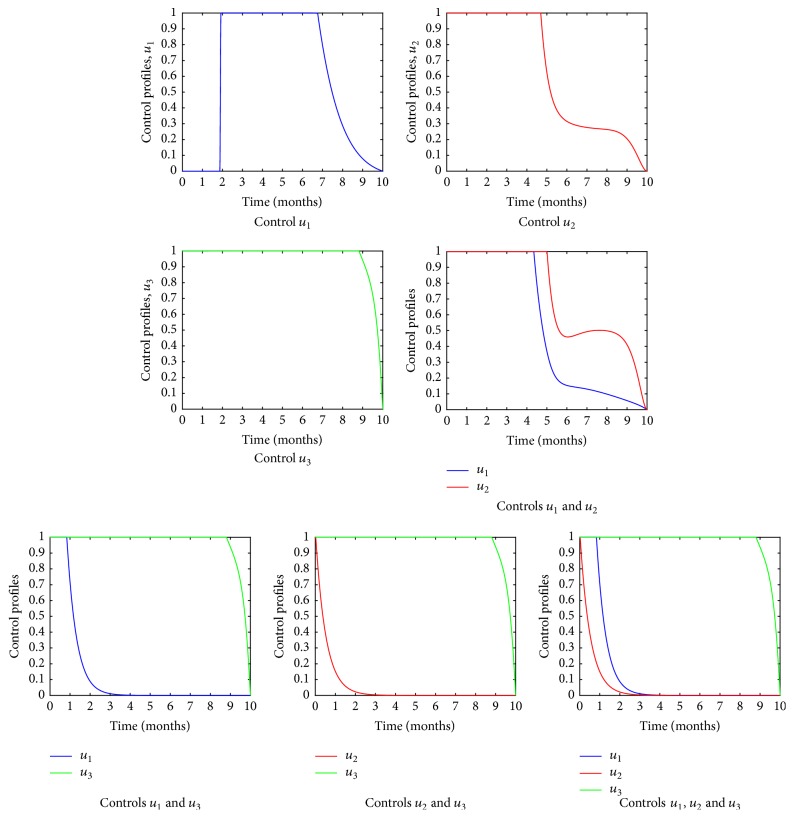
Simulated control strategies.

Figure 2 represents the various control strategies. It is evident that the control *u*_1_ remains at the maximum for the first two months and drops to zero onward, while control *u*_2_ remains at maximum for the first four and a half months and then drops to 30% the sixth and the ninth months and drops to the minimum after the 10th month. In addition, the control strategy *u*_3_ remains at a maximum for the first ninth months, only dropping to a minimum at the tenth month. From these results, we can see that protease inhibitor can be administered for a longer period of time.

**Figure 3:**
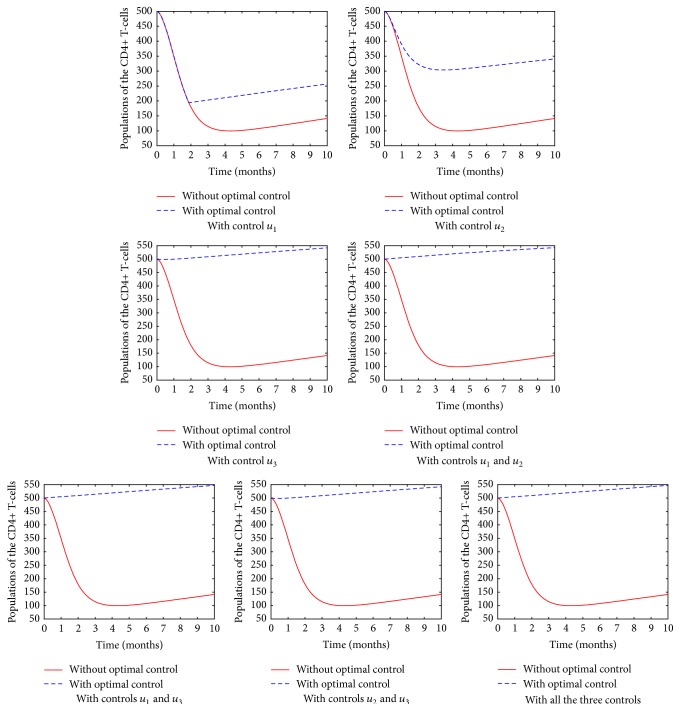
The population of the CD4^+^ T-cells in various control strategies.

Figure 3 shows the population of the CD4^+^ T-cells in different treatment strategy. In all the cases, it is evident that the introduction of the ARTs plays a significant role as far as controlling HIV is concerned. Nonetheless, it is clear that when fusion inhibitor (*u*_1_) is used without other controls, the number of CD4^+^ T-cells reduces significantly and a longer time is taken before the number increases. In particular, the drug effectiveness seems to be felt after the first two months. We interpret the results to mean that it is difficult to control the HIV virions by targeting their cell-entry mechanism. The use of protease inhibitor, however, leads to an increase in the number of the CD4^+^ T-cells. In addition, it is evident that a combination of the three drugs evokes a more pronounced CD4^+^ T-cells increase than in monotherapy or combination of two drugs.

It is important to point out that CD4^+^ T-cell responses in number of cells gained were similar for patients treated with combination of two drugs therapies and patients treated with combination of three drugs therapies.

**Figure 4:**
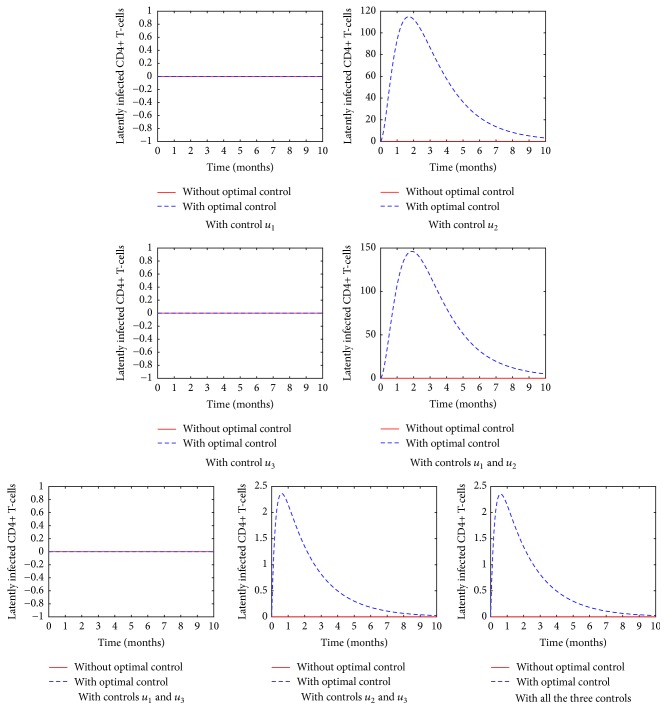
The population of the latently infected CD4^+^ T-cells in various control strategies.

Figure 4 presents the dynamics of the latently infected cells after the introduction of the various control strategies. It is evident that the latently infected cells are produced after the introduction of reverse transcriptase inhibitor to an HIV infected cell. Since the latently infected cells do not produce infectious virions, it is important to administer RTIs to an infected person. This will reduce the number of virions producing cells.

**Figure 5:**
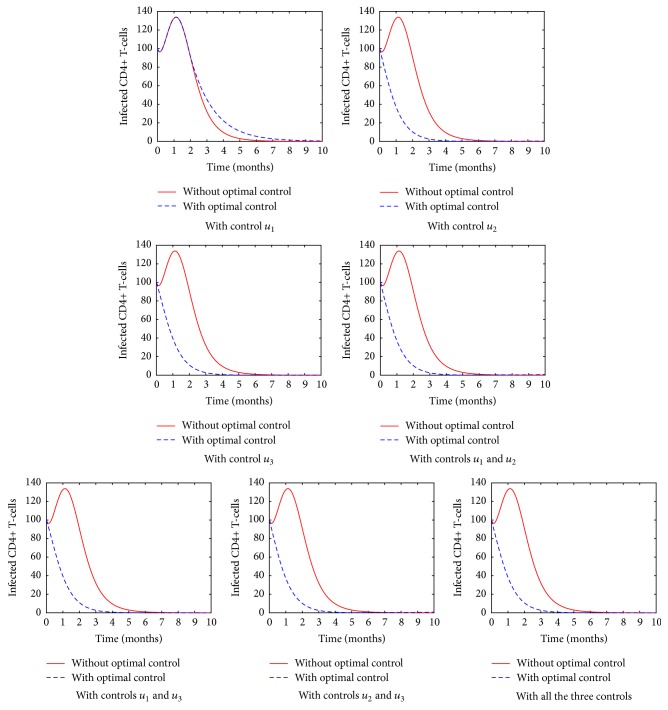
The population of the infected CD4^+^ T-cells in various control strategies.

**Figure 6:**
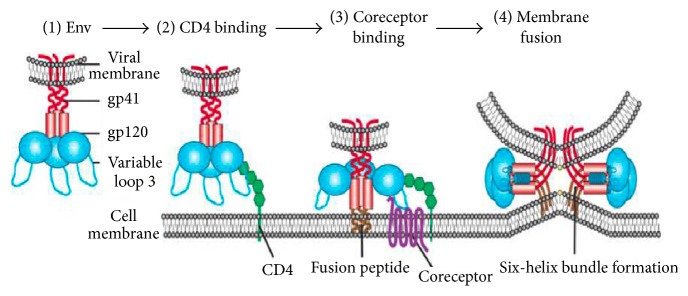
HIV entry mechanism [13].

Figure 5 shows the change in the population of the infected CD4^+^ T-cells with time in different control strategies. From the simulated results, we see that use of ARTs plays a fundamental role, especially in controlling the rate of infection. Nonetheless, when the fusion inhibitors are introduced in the body, the number of the infected cells still increases for the first few months. This clearly shows that it is very difficult to control the HIV virions at the entry level. The reason would probably be based on the fact that HIV uses a complex series of steps to deliver its genome into the host cell cytoplasm while simultaneously evading the host immune response as shown in Figure 6.

**Figure 7:**
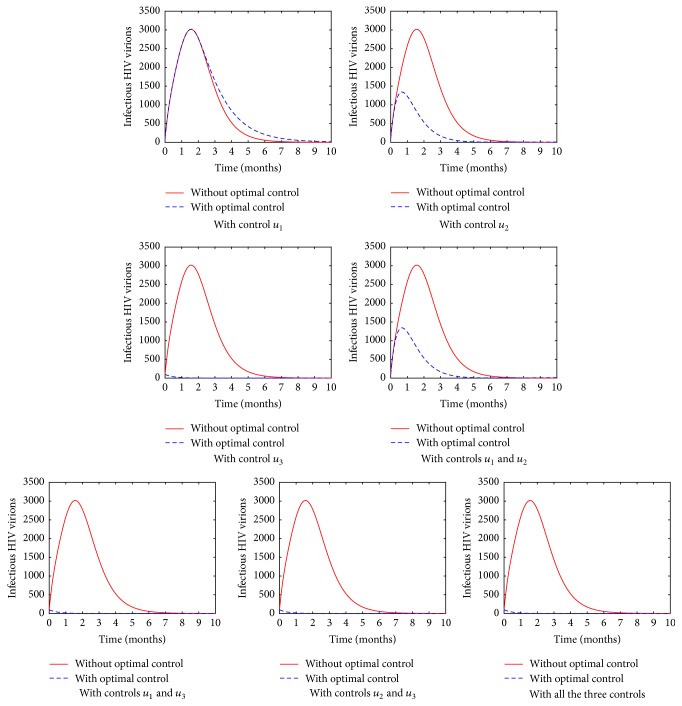
The population of the HIV virions in various control strategies.

Figure 7 shows the change in the population of the HIV virions in different drug combination(s). It is evident that controls *u*_1_ and *u*_2_ are not as very effective as PIs in controlling viral progression. In particular, there is no significant difference when the control *u*_1_ is used and when no control is used at all. Researches such as [20] suggest that viruses blocked by entry inhibitors such as the fusion inhibitors are likely redistributed to plasma, where they artificially increase the number of HIV virions. This may probably be the reason why there is an indication of having high number of viral loads even when control *u*_1_ is applied. In addition, the fusion inhibitor prevents the entry of the virions unlike the other two drugs that allow the entry of the HIV virions into the cells, confirming the absorption effect. Simulated results shows that protease inhibitor plays a significant role in reducing viral progression and it is the best single drug in use for viral suppression. This is in agreement with some of the works done in the field of in vivo HIV dynamics which have concluded that protease inhibitors are more effective than reverse transcriptase inhibitors and fusion inhibitors in terms of viral load reduction in HIV infected patient [21–23]. The simulated results also emphasize the importance of using a combination of the various ARTs when treating HIV.

**Figure 8:**
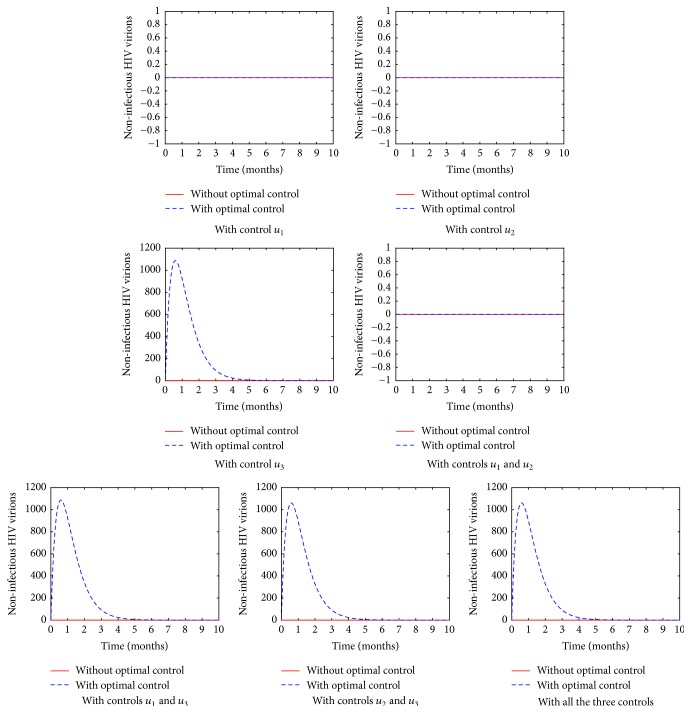
The population of the noninfectious HIV virions in various control strategies.

From Figure 8, it is evident that noninfectious viruses are produced after the introduction of the protease inhibitor in the body. Introduction of PIs to HIV infected cells generates a pool of immature HIV virions; this leads to the transfer of noninfectious virus across the virological synapse. This therefore implies that the virus produced will not infect more susceptible CD4^+^ T-cells.

**Figure 9:**
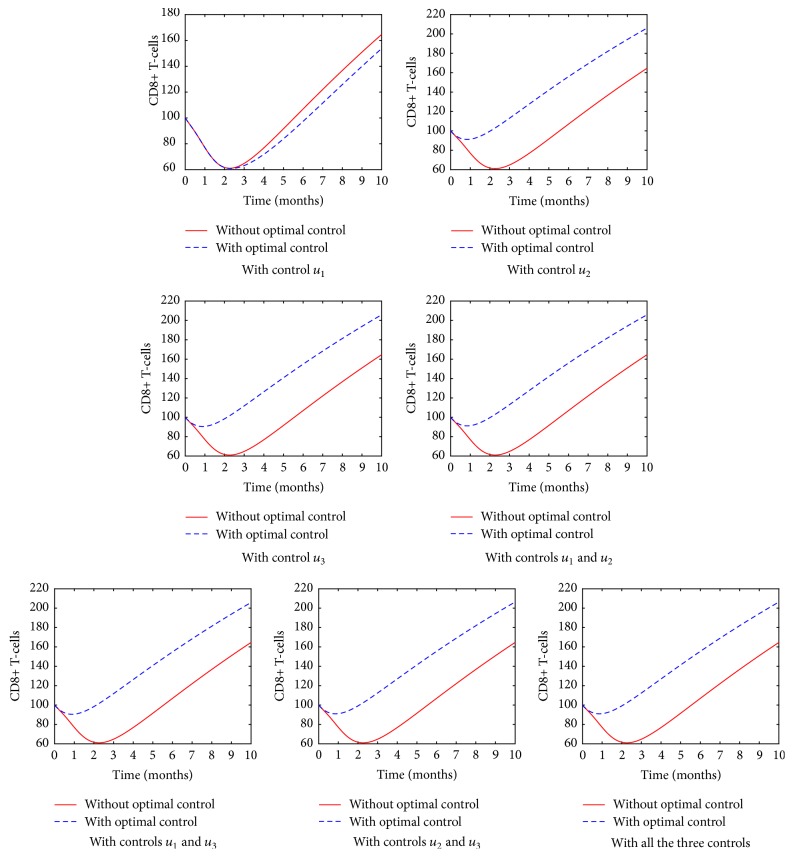
The population of the CD8^+^ T-cells in various control strategies.

Figure 9 shows the population of the CD8^+^ T-cells in different treatment strategy. Both the RTIs and PIs cause a substantial increase in the population of the CD8^+^ T-cells in HIV infected patients. However, it is evident that as much as these two drugs plays a major role, the combination of all the three controls produces a higher immune system reconstitution with sustained increases in circulating number of CD8^+^ T-cells.

**Figure 10:**
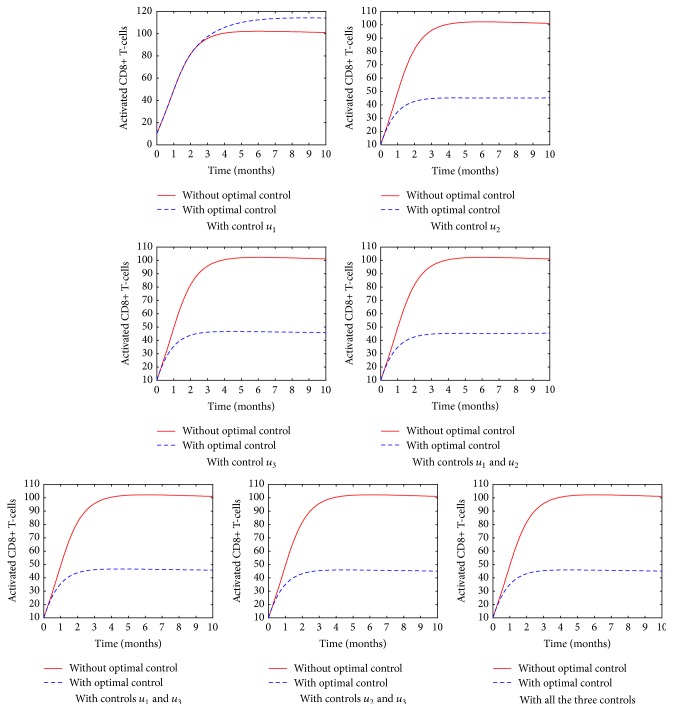
The population of the activated CD8^+^ T-cells in various control strategies.

Figure 10 shows the population of the activated CD8^+^ T-cells. The activation process plays a major role in controlling the HIV virus particles. This is because the cells fight, destroy, and kill the infected CD4^+^ T-cells. This in turn reduces the number of HIV virions produced. From the simulated results, it is evident that, after the introduction of the ARTs, the number of activated CD8^+^ T-cells reduced significantly. The reduction may be attributed to the reconstituted immune system or due to the reduction of the retroviral activity on the cells [24]. However, the question we need to ask ourselves is whether this reduction has any clinical benefit. In the future, it is important to analyze the clinical benefit accrued from the reduction of the CD8^+^ T-cells activation process.

## 7. Conclusion

In this paper, we have analyzed a seven-dimension in vivo HIV model with inclusion of three drug combinations, that is, FIs, RTIs, and PIs. Optimal control theory is applied to determine the optimal treatment regime. The study applied Pontryagin's Maximum Principle in deriving the conditions for optimal control, which maximizes the objective function. The systems of ODEs, the state system, and the adjoint system were solved numerically by both forward and backward Runge-Kutta forth-order scheme. Results from the numerical simulations show that FIs and RTIs should be used within the four months and later the doctors should change the drugs and introduce another type, whereas the PIs can be used for a longer period of time without necessarily leading to major side effect. However, the inferiority of monotherapy compared with combination of therapies has been observed in the simulated result, especially in suppression of viral replication, CD4^+^ and CD8^+^ T-cells reconstitution, and controlling disease progression.

ARTs have been seen to play a significant role as far as viral suppression is concerned. Therefore, they should be recommended for all patients immediately after one is diagnosed as HIV-positive regardless of the CD4^+^ count. This supports the guidelines by WHO. However, the simulated results suggest that PI is possibly the best single drug and fusion inhibitor is the worst drug in terms of viral load and infected cells reduction. From the results, we recommend that RTIs be used as initial therapy for HIV. FI should be introduced to the patient after the RTIs but should never be used alone.

In the future, it is important to develop the model in such a way that it brings out the relationship between the number of the CD8^+^ T-cells and the CD4^+^ T-cells produced in the thymus.

## Figures and Tables

**Table 1:** Variables for HIV in vivo model with therapy.

Variable	Description
*T*(*t*)	The concentration of the noninfected CD4^+^ T-cells per cubic millimetre at any time *t*
*I*(*t*)	The concentration of the infected CD4^+^ T-cells per cubic millimetre at any time *t*
*I*_*l*_	The concentration of latently infected CD4^+^ T-cells per cubic millimetre at any time *t*
*V*(*t*)	The concentration of HIV virions, copies/mL, at any time *t*
*V*_*n*_(*t*)	The concentration of the immature noninfectious virions, copies/mL, at any time *t*
*Z*(*t*)	The concentration of the CD8^+^ T-cells per cubic millimetre at any time *t*
*Z*_*a*_(*t*)	The concentration of the activated CD8^+^ T-cells per cubic millimetre at any time *t*

**Table 2:** Parameters for HIV in vivo model with therapy.

Parameter	Description
*λ*_*T*_	The rate at which the noninfected CD4^+^ T-cells are produced per unit time.
*μ*_*T*_	The rate at which the noninfected CD4^+^ T-cells decay.
*χ*	The rate at which the CD4^+^ T-cells are infected by the virus.
*μ*_*I*_	The death rate of the infected CD4^+^ T-cells.
*μ*_*I*_*l*__	The death rate of the latently infected CD4^+^ T-cells.
*ε*_*V*_	The rate in which HIV virions are generated from the infected CD4^+^ T-cells.
*μ*_*V*_	The death rate of the infectious virus.
*μ*_*V*_*n*__	The death rate of the noninfectious virions.
*α*	The rate at which the infected cells are eliminated by the activated CD8^+^ T-cells.
*λ*_*Z*_	The rate at which the CD8^+^ T-cells are produced per unit time.
*μ*_*Z*_	The death rate of the CD8^+^ T-cells.
*β*	The rate at which the CD8^+^ T-cells are activated by the presence of the virus and the infected CD4^+^ T-cells.
*μ*_*Z*_*a*__	The rate at which the activated defense cells decay.

**Table 3:** Control variables for HIV in vivo model.

Control variable	Description	Purpose
0 ≤ *u*_1_ ≤ 1	Fusion inhibitors	Are a class of antiretroviral drugs that work on the outside of the host CD4^+^ T-cell to prevent HIV from fusing with and infecting it.

0 ≤ *u*_2_ ≤ 1	Reverse transcriptase inhibitors	Are a class of antiretroviral drugs used to treat HIV infection by inhibiting the reverse transcription process.

0 ≤ *u*_3_ ≤ 1	Protease inhibitors	Are a class of antiviral drugs that are widely used to treat HIV/AIDS by inhibiting the production of protease enzyme necessary for the production of infectious viral particles.

**Table 4:** Parameters and controls for HIV in vivo model with therapy.

Parameters	Value	Source
*λ*_*T*_	10 cell/mm^3^/day	Nowak et al. [8]
*μ*_*T*_	0.01 day^−1^	Srivastava and Chandra [9]
*χ*	0.000024 mm^3^ vir^−1^ day^−1^	Alizon and Magnus [10]
*μ*_*I*_	0.5 day^−1^	Wodarz and Nowak [11]
*μ*_*I*_*l*__	0.5 day^−1^	Wodarz and Nowak [11].
*ε*_*V*_	100 vir. cell^−1^ day^−1^	Estimate
*μ*_*V*_	3 day^−1^	Mbogo et al. [2].
*μ*_*V*_*n*__	0.06 day^−1^	Estimate
*α*	0.02 day^−1^	Arruda et al. [12]
*λ*_*Z*_	20 cell/mm^3^/day	Arruda et al. [12]
*μ*_*Z*_	0.06 day^−1^	Arruda et al. [12]
*β*	0.004 day^−1^	Arruda et al. [12]
*μ*_*Z*_*a*__	0.004 day^−1^	Arruda et al. [12]
*u*_1_	0-1 variable	Estimate
*u*_2_	0-1 variable	Estimate
*u*_3_	0-1 variable	Estimate

**Table 5:** The initial values for the variables for HIV in vivo model.

Variable	Values
*T*(*t*)	*T*(0) = 500 cell/mm^3^
*I*(*t*)	*I*(0) = 100 cell/mm^3^
*I*_*l*_	*I*_*l*_(0) = 0 cell/mm^3^
*V*(*t*)	*V*(0) = 100 virion/mm^3^
*V*_*n*_(*t*)	*V*_*n*_(0) = 0 virion/mm^3^
*Z*(*t*)	*Z*(0) = 100 cell/mm^3^
*Z*_*a*_(*t*)	*Z*_*a*_(0) = 10 cell/mm^3^
